# Observations on Fever

**Published:** 1828-04

**Authors:** 

**Affiliations:** Alnwick.


FEVER.

*Observations on Fever.* Addressed to a Medical Friend, by Dr. Bow, of Alnwick.

(Continued from page 194.)

In this stage, the two constant and prominent symptoms are increased heat of the body and increased action of the sanguiferous system; but whether increased heat of the body precede increased action of the sanguiferous system, or vice versâ, is a point which has not been sufficiently attended to, and that, perhaps, because the practitioner is seldom called on until the fever is fully developed. I may say, however, that it is generally believed and inculcated that increased action of the sanguiferous system is the prior symptom, and also that it is generally believed that this increased vascular action is the cause of the heat. I have been subject to fever resembling ague, and in my own case I can assert that there were sensations of heat on various parts of the body before I could recognise any alteration of pulse; but I must observe that I have had only one paroxysm since it occurred to me to watch the priority of these symptoms, therefore I shall lay no stress on my own case. In various authors we meet with remarks touching this point. Dr. HAMILTON, in his observations on the seats and causes of diseases, says that increased heat of the body, or at least of particular parts of it, is sometimes felt independent of increased action of the sanguiferous system. And still more to the purpose is the testimony of Dr. CARMICHAEL SMYTH. Whilst this physician was inquiring into the nature of the fever which attacked the Spanish prisoners at Winchester in 1780, he was himself attacked, and, in the description of his own case, he says that, after a long exposure to the contagion, although he had felt no inconvenience within the house (where the sick were), he afterwards found himself very giddy, with a considerable degree of nausea. These symptoms, however, soon went off: he ate his dinner as usual, and went to bed seemingly in perfect health; but, about the middle of the night, he awoke from sleep with symptoms of the most violent fever, the oppression of the precordia exceeding all description, whilst the sensation of heat gave him the idea of liquid fire spreading from his stomach across his breast, along the course of the pectoral muscle, and down the inside of his arms to the extremities of his fingers. But, notwithstanding these dreadful sensations, his pulse was regular, and the frequency of it by no means in proportion to the degree of heat; but this was not the case in after periods of the disease.

If increased heat be sometimes felt independent of increased action of the sanguiferous system, it is plain that the latter cannot be looked upon as the cause of the former, and therefore we must look for some circumstance, the effect of primary disorder, which must intervene, and act as the immediate cause of excitement.

I think I can trace this cause, and in it I can see nothing but what is natural and simple,—to an effect of impaired energy of the brain, which I purposely omitted before, to save repetition. We look upon the effect of the remote cause of fever to be impaired energy of the brain, and, from the very sudden invasion of fevers in many cases, and from the very sudden effect of certain gases when inhaled, we are led to believe that the remote cause acts immediately on the extremities of the nerves with which it comes in contact. We recognise this impaired energy by the symptoms to which it gives rise, and if slightly impaired energy cause listlessness, dejection of spirits, and muscular debility, it is but reasonable to conclude that the function of secretion must also in some manner be effected: nor is this a gratuitous assumption, for in the cold stage of fever there is evidence enough to prove it. That the secretions in general are deficient in quantity during the cold stage of fever, no one will deny; yet it may be said that as the capillary arteries are less active in that stage, the secretions may be defective in quantity owing to the want of material for their production; but is that which is secreted perfect in quality? The white and clammy state of the tongue shows an altered condition of the mucous products; the loathing of food shows that the gastric juice is not as it ought to be; the appearance of the stools points to an imperfect state of the bile. If a product of secretion in any one part of the body be altered in its nature in consequence of impaired energy of the brain, we may or must infer that the products of secretion throughout the body will also be affected more or less from the same cause. And when we consider that there is no part of the body in which the operation of secretion is not going forward, and that each and all of its products may become sources of irritation, we cease to wonder that fever should be a disease "affecting the whole system."

In the first stage of fever, the secretions must be regarded as deficient not only in quantity, but also in quality; and these defective secretions again can only be looked on as foreign matter, which after a time must irritate the nervous extremities with which it is in contact, and it is this irritation which, in my humble opinion, determines the hot stage. Diminished sensibility is observed to follow impaired nervous energy; it is therefore reckoned amongst the phenomena of the first stage; and the duration of the first stage is in every instance longer or shorter according to the degree of diminution of sensibility, or the morbid state of the secretions. If sensibility be but little diminished, the cold stage will be of short duration, for in that case the impression from the imperfect secretion is soon conveyed to the sensorium, and the brain being thus roused, nervous influence in greater force is diffused throughout: if, however, sensibility be greatly diminished, then will the cold stage continue until the secretions acquire vitiation enough to irritate. "The sensibility of the skin differs very much in its different parts, but in its general extent it may be considered as possessing the most acute degree of feeling of any of the structures of which the body is composed;"* and it is on that account that the symptoms of reaction first appear on the surface. It is the acute degree of feeling which the skin possesses in its general extent, which gives the disease the form of fever; for were sensibility confined to a part of small extent, the determination of nervous influence to that part would be in force sufficient to excite inflammation; whereas, owing to the general extent of sensibility, the irritation becomes general, and consequently the determination of nervous influence must be general also. But as hereditary taints, former disease, or intemperate habits, may have impaired the functions of some organs, or even altered the structure of others, we frequently meet with topical inflammations occurring at the onset of the second stage, and which in many cases completely alter the form of the disease. The peculiar state of an organ, whether we regard its structure or function, may render it more liable than other parts to be affected by causes operating upon the system at large: for example, the fluid destined to lubricate the pleura may become sooner or later more completely defective than the secretions of other parts, and, by its irritating nature, cause a determination of nervous influence to that texture, before the imperfectly secreted products of the skin can, by irritation, elicit a determination to the surface; or, though this fluid may not become more defective than other secretions, the pleura, owing to former lesion or other causes, may be more sentient than when perfectly sound, and consequently more obnoxious to irritation; in either of which cases inflammation of that membrane will manifest itself at the commencement of or during the three first days of the second stage. On the other hand, a part may be defective in such a manner as to be less sentient than when sound, or than other parts, and which, during the three first days of the second stage, may attract no particular attention; but, when nervous energy in general begins to decline, then will the energy of this part decline in a greater ratio, or, in other words, this part will lose tone sooner than other parts: hence will follow that topical inflammation termed "subacute inflammation," being sanguineous congestion owing to want of energy in the part. To illustrate more fully,—there is a state in which the system is rendered highly susceptible of impression from a typhoid miasm, attended with local circumstances which dispose the disorder to assume an inflammatory form: I mean the puerperal state. The excitement attending parturition soon gives way, and is followed by the debility of exhaustion,

* Bostock.

in which condition the slightest morbific agent is sufficient to involve the system, which but a few hours before might have opposed a causation of tenfold greater force.

The disease is ushered in by a cold fit, during which the secretions are imperfectly produced; and as with puerperals there is one texture, the peritoneum, which may be regarded as in a morbid state, we at once can pronounce the cause of its being the seat of inflammation. The peritoneum, during its extension, must have its vessels and nerves either enlarged or multiplied, or both: therefore, when relaxed, it must for a time be supplied with a greater force of nervous influence and a greater quantity of blood than is necessary: hence it is more sensitive, and the exhalation from its surface more abundant, than necessary. In this condition is the peritoneum more liable to be irritated; but the superabundant secretion from its surface becoming imperfect, in common with the other secretions of the body, acts like foreign matter applied to its whole extent, and involves it in inflammation before the irritation on the other textures of the body can determine general reaction. Indeed, in some cases of the true puerperal fever, where the severity and extent of the peritonitis are great, we may look in vain for general reaction; the determination of nervous influence to the irritated or inflamed surface being so powerful that nothing can divert its course: hence the languid energy in other parts, giving rise to the "pellucid eye, the pale and shrunk countenance, the indescribable expression of anxiety."

Before I leave this part of my subject, let me call your attention to the nature of the heat, or rather the sensations which accompany the heat evolved in fevers. I am sure you will at once agree with me, that it is a heat depending on irritation in the part in which it is felt; and, as a proof of this, it may only be necessary to quote a few of the adjectives employed by authors to denote that it is a heat of no common kind: for instance, we have ardent, pungent, burning, penetrating, and a heat like liquid fire. As the heat in fevers cannot be the effect of increased action of the sanguiferous system, inasmuch as the former has been observed independent of the latter, neither can the inordinate action of the heart be the effect of the heat; for, in like manner, inordinate action of the heart has been felt unaccompanied by heat such as is experienced in fever. When reaction in general is taking place in consequence of irritation from the imperfect secretions throughout the body, and particularly of the skin, there can be little doubt but that the heart will suffer an increase in its action ; but I suspect that the inordinate action is attributable to the absorption and commixture with the blood of the imperfect products of secretion.

In this stage (of excitement) commence those symptoms which led to the idea that fever is an effort to expel something hurtful, either ingenerated or introduced from without. Such was the opinion of HIPPOCRATES, of GALEN, and of SYDENHAM; and it is a pity that this hypothesis, which stood the test of so many ages, should have been at length neglected, or that physicians should have been blinded by the ingenious but unsubstantial speculations of later days; for, although the disease does not originate from a morbid change either of the blood or fluids secreted from it, yet a change in the latter does take place in consequence of a prior morbid action; and it is this change, and the action which follows as effects, that give form to the disease, and entitle it to be called Fever.

The doctrine of the Greek school was derived from observation, and it is one which would naturally follow, from "beholding a violent commotion in the system followed by an evacuation from the skin and kidneys, with which the paroxysm terminated." It would seem that one objection to this hypothesis must fall to the ground if we could in any plausible manner explain how morbific matter is generated internally; for Dr. Good says that there are many fevers produced evidently by cold, fear, and other excitements, as well mental as corporeal, in which most certainly there is no morbific matter introduced, and wherein we have no reason to conceive there is any generated internally; while the disease, limited perhaps to a single paroxysm, closes nevertheless with an evacuation from the skin or kidneys.

Now, in such fevers, the first effect of the remote cause is impaired nervous energy; there is a cold stage, during which the secretions are disturbed, whereby we have great reason to believe that imperfect products are formed, which, though not morbific, are morbid and generated internally, and which, being absorbed, are thrown from the system through the skin, kidneys, or bowels. As fevers have been known to have been suddenly cured by a hemorrhage so moderate in quantity as to be incapable of carrying out any considerable portion of matter diffused over the whole mass of blood, and as Dr. Good cannot conceive how such diffused matter can collect itself at a focal point, or pass off at a single outlet, he considers the hypothesis incorrect. When, however, foreign matter is purposely introduced into the system, we are witnesses to the commotion it excites, and the animal only recovers after some critical discharge. With regard to not conceiving how offending matter can collect itself at a focal point, or pass off at a single outlet, I would ask if it were more preposterous to suppose the blood-vessels endowed with a power of casting from them that which is offensive, than absurd to give to the lacteals the power of selection or rejection; yet no one disputes their possession of such power. That there is a law in the animal economy which determines offending matter to the points irritated, I think cannot be denied, for in our daily practice we behold the effects of its operation; but to explain how it operates may be a difficult task. If, from whatever cause, menstruation be suddenly checked, how often then do we see erysipelatous inflammation follow an incautious exposure to cold; and is not erysipelatous inflammation in every instance caused by a deposition of irritating matter from the blood. It is said that wounds of the pericranium are oftener followed by erysipelas than other wounds, but it does not follow, as is supposed, that erysipelas obtains in consequence of the pericranium being wounded. In most cases, a blow sufficient to wound that membrane is concussive enough also to derange the operations of the brain: hence for a time the secretions are imperfectly produced,—they are absorbed and deposited round the wound, the point irritated. If the concussion be violent, and the operations of the brain for a longer time deranged, then the secretions become so vitiated that, before they are absorbed, they irritate generally, by which reaction is brought about, and the absorbed offending matter is at length gradually discharged from the wound in the form of pus. When concussion proceeds from violence not producing lesion, then is there fear of irritation or inflammation of the brain or its membranes; for, there being no wound or external irritation to which the absorbed offending products can be determined, they are attracted towards the brain, the seat of the injury, and there produce irritation and inflammation: or, as in fevers, it may be thrown off by a critical discharge. Indeed, I very strongly suspect that that form of fever denominated "sympathetic" is also an effort of the system to throw off something hurtful generated internally; or, I would rather say, is produced by offending matter circulating with the blood. For, if irritation from an external cause be continued, nervous influence is directed in particular to that part, and inflammation is the consequence; but whilst so great a force of influence is directed suddenly to one point, other organs or textures are, on that account, proportionally deprived of it, and consequently the secretions of such parts become imperfect, are absorbed, and give rise to the fever. Here there is offending matter which must be thrown off. If it be thrown off by the bowels, kidneys, or skin, we have that termination of inflammation termed "resolution;" but it oftener happens, where the inflammation is severe, that the offending matter is determined to the inflamed point, and deposited there in the form of pus: hence suppuration ought to be regarded rather as the crisis of the fever, than a termination of inflammation. Is not menstruation itself a strong proof that the system has the power of collecting offending matter at a focal point, or of discharging it at a single outlet; and that this power is not confined to one part, or particular structure, is evident from the fact that, by irritation, the menstrual discharge can be diverted from its natural channel. We have many instances on record wherein it has made its exody by an ulcer on the leg or arm. If irritation, then, such as is produced by an ulcer on the leg or arm, can withdraw a discharge from its natural channel, need we be surprised that, when offending matter is circulating with the blood, that irritation should determine its outlet?

The increased energy which marks the second stage generally begins to decline on or after the third day of it; after which time (provided there be no local determination of nervous influence, caused by an irritation greater than that which a few leech-bites might create,) I have frequently seen fevers yield to an hemorrhage caused by two or three leeches applied as near as possible to the same spot; or to the hemorrhage from one bite promoted by a cupping glass. If benefit be therefore derived from the loss of so small a quantity of blood, we must impute the favorable result to the nature, and not the quantity of blood lost. Indeed, the effects of leeches at a certain period in fevers, have often been so favorable as to excite amazement even in the practitioner; and, in some cases, so efficacious has the operation been, as to induce some to believe leeches possessed of endowments beyond drawing blood. If we examine the action of some of our favorite remedies in fever, we may trace the benefit derived from them to their establishing an outlet for offending matter. Cathartics, administered daily during the first stage, not only empty the gut, but, by keeping up increased intestinal action, they secure a drain or outlet for offending matter; and hence the cause why the early administration of cathartics, for the most part, insure a short second stage.

Although I set out with the determination of meddling as little as possible with the opinions of others, I cannot help stating that I think it is the presumed *modus operandi* of cathartics that has led to the idea that fever, in its first stage, is an affection of excitement. The evacuant plan, as it has been called, is supposed to be productive of benefit by clearing the gut, and by lessening the volume of the circulating mass. After a brisk cathartic given in the first stage, the patient may, and often does, feel renovated, while a person in health, after a similar dose, feels exhausted: hence it is argued, if fever be an affection otherwise than of excitement in its first stage, the patient's feeling, after a cathartic, would be that of increased weakness. Impaired energy of the brain from a febrific miasm, does not imply an exhaustion of nervous influence, such as follows great bodily fatigue; the sensibility and energy of the brain are obtruded, by which it cannot sufficiently acknowledge the usual impressions which regulate its operations: requiring, therefore, stronger impressions to rouse it to action, it is in a manner paralysed. The first effect of a cathartic is irritation of the gut, and this impression, being stronger than that produced by the natural irritants of the canal, is acknowledged. The brain being thus roused, nervous influence, though in particular directed to the gut, is diffused throughout: hence the patient feels himself renovated, and it is diffused throughout, because the brain, being roused, becomes again more sensible to the natural impressions throughout the body. It is this feeling of renovation after a purgative in the first stage, conceiving it to be produced by a diminution of the circulating mass, which I think, with great deference to so high authority, led Dr. Armstrong to view fever as an affection of excitement in its first stage, and to consider the nervous appearances, even from the first attack, as only secondary to vascular disorder. That vascular disorder will occasion nervous appearances, cannot be doubted; for we know that the vascular and nervous systems are positively dependent on each other; but that the vascular system should become deranged, unless as an effect of nervous derangement, I cannot believe. I have imputed rigors to sanguiferous congestion, and therefore that nervous symptom I conceive to be secondary of vascular disorder; but I hold that retrocession of blood from the surface and internal accumulation can only happen in consequence of nervous disorder. Dr. Armstrong maintains that the debility in the first stage of typhus is chiefly dependent upon preternatural accumulation of blood in the vessels about the head, heart, liver, and other internal parts, and that this debility is only apparent. But general internal accumulations of blood can only happen in consequence of debility and lessened irritability of the vessels themselves. In the first stage of fever, the state of the surface of the body shows that debility extends to the small vessels of the skin, which, if it were confined to those vessels alone, instead of manifesting itself in an empty state of the vessels, would do so in a state of engorgement; for the heart and larger arteries, continuing in a state of vigor, would propel the blood into them as usual, whilst they wanted the power to forward it. This vascular debility, however, being general, atmospheric pressure determines the internal accumulations; therefore these accumulations, however much they may increase debility, are themselves depending on it.

With regard to the debility of the body being apparent, it is, in my opinion, as direct as if it were the consequence of exhaustion; for the debility in the first stage of fever depends on nervous influence being withheld; debility from fatigue depending on its being exhausted; the cause of debility in both instances being the want of nervous influence. Yet there is a difference, and a very material one in a practical point of view, and that difference lies in the state of the brain. The debility depending on influence being withheld may be soon removed by whatever rouses the brain into action, while that depending on exhaustion is increased by attempts to rouse the energy of the brain. When general reaction takes place, the internal accumulations are removed, and still the debility continues.

Having imputed the debility to vascular disorder in the first stage, Dr. Armstrong thinks it necessary to blame vascular disorder also for the second stage, although the disorder in the first stage plainly consists of want of excitement in the heart and arteries, and in the second stage of over-excitement. It certainly in both stages depends on the same cause, viz. diminished transmission of nervous influence.

This cause of debility may seem as paradoxical as vascular disorder, since in the first stage nervous influence is evidently withheld, and in the second stage it is as evidently transmitted in greater than natural force. But, though thus transmitted from the brain, it is still withheld from the muscular apparatus; for, owing to the superior sensibility of the skin, the surface of the body is the part first irritated by its imperfectly secreted products, and consequently the determination of influence is to the surface: for the same reason is it withheld from the gut, and hence the constipation which generally accompanies the second stage.

